# Bacteriology in Relation to Dental Caries

**Published:** 1918-11

**Authors:** 


### BACTERIOLOGY IN RELATION TO DENTAL CARIES

If an attempt is made to bring some order out of the chaos in which the scientific aspects of dental caries seem to be involved, it appears not unlikely that several factors of unlike character are concerned. They have been fancifully represented as "defensive" on the one hand, and "attacking" on the other. Among the latter group, bacteria and carbohydrates probably deserve the foremost attention in the study of the etiology of decay of the teeth. The pioneer studies of Miller on the bacteriology of the mouth gave great weight to his views; hence even today his conception of dental decay still is prominent. Miller's idea was that lactic acid or similar acids produced by bacterial fermentations of the carbohydrates in the mouth are responsible for the decalcification of the enamel, and that the exposed dentin is further destroyed by the proteolytic ferments of the same or similar bacteria. But as Meyer[1] has pointed out in a review of the present status of dental bacteriology, neither Miller nor any of the subsequent investigators has succeeded in finding any organisms strictly specific for caries.

[1] Meyer, K. F.: The Present Status of Dental Bacteriology, Jour. National Dental Assn., 1917, **4**, 966.

The medical profession has scarcely yet become familiar with the more recent studies of Kligler[2] in this country on the microbiotic flora of the normal mouth and also the changes observed when dental caries occur. All the families of bacteria are represented in the healthy oral cavity. Most predominant are the representatives of the cocci and streptothrica or trocomycetes group. On the healthy teeth the types of organisms and their relative abundance remain apparently constant, although with the changing conditions in the mouth there is a variation in the total numbers of bacteria. From the available data Meyer has gathered that the number under ordinary conditions in 1 mg. of deposit consists of about twenty-five million organisms, of which one million can be cultivated. In dirty mouths the counts were about twice as high. The growth of oral micro-organisms is just as much influenced by changes in the mouth as is the case for bacterial activity and growth in the soil or in the intestinal tract. The total number of bacteria increases during the night and immediately following a meal. Miller adds that the stagnant conditions in the mouth inducing concentration of fermentable carbohydrates causes a shift in the general relationship of the types to one another, a decrease of the cocci and increase of the forms characteristic for early stages of decay. The number of bacteria on unbrushed teeth were about four times as great as those on brushed teeth. Three times the number of organisms were found in dental deposits immediately after meals, in contrast with that present before meals. Brushing of the teeth or chewing tobacco removes about three-fourths of the total number of bacteria.

The first stages of caries which, in the judgment of Kligler, can be considered a specific infection, show enormous counts and an entirely different flora from that of

[2] Kligler, I. J.: Jour. Allied Dental Soc., 1915, **10**, 141, 282, 314.

normal teeth. With the progress of decay the character of the dominant micro-organisms changes. As Meyer has summarized it, in comparison with the clean and normal mouth, there is a decrease of the streptococci in all stages of decay and a marked increase in the acidific bacilli. The medium of the mouth is poor in proteins and usually contains carbohydrates; therefore, it is unsuitable for proteolytic organisms. Only in such portions of the buccal cavity, like decayed teeth, where symbiosis with strongly aerobic organisms and cumulation of nitrogenous material produces a suitable soil, can strongly proteolytic anaerobic bacteria exist and develop.

Let it be clearly asserted that the relations of cause and effect are by no means clearly demonstrated in the bacteriologic studies of decay made up to this time. But if one is justified, from such somewhat indefinite findings as are presented, in assuming that oral micro-organisms are in some way or to some degree responsible for dental decay, it is logically in order to attempt to eliminate the causal agents. As Meyer indicates, sterilization by chemical means is impossible; and the use of such procedures may actually be harmful to the tissues of the mouth cavity. There is something both novel and modern in the further suggestion of studies along chemotherapeutic lines leading to the possible discovery of highly parasitotropic and slightly organotropic substances in an attempt to gain mastery over the objectionable micro-organisms. The further existence of possible foci of systemic infection in the mouth cavity opens up still different problems. It seems likely, indeed, that bacteriology and the dental clinic will become more closely related to each other in the immediate future.—*Journal A. M. A.*, September 21, 1918.

